# For Pediculi Pubis

**Published:** 1891-03

**Authors:** 


FOR PEDICULI PUBIS, rub the affected part witb a piece of flannel wet with the following mixture :

Salicylic acid, 2 or three parts.
Toilet vinegar, 25 parts.
Alcohol (80 percent.) 75 parts.

One application is usually sufficient.

